# A New Analytical Method to Quantify Ammonia in Freshwater with a Bulk Acoustic Wave Sensor

**DOI:** 10.3390/s22041528

**Published:** 2022-02-16

**Authors:** Vera Lúcia M. Antunes, Maria Teresa S. R. Gomes

**Affiliations:** CESAM, Department of Chemistry, University of Aveiro, 3810-193 Aveiro, Portugal; luz.antunes25@gmail.com

**Keywords:** ammonia, piezoelectric quartz crystal, acoustic wave sensor, freshwater, amines

## Abstract

A new method to analyse ammonia in freshwater, based on a piezoelectric quartz crystal coated with the metalloporphyrin chloro[5,10,15,20-tetrakis(pentafluorophenyl)porphyrinato] manganese(III) is presented. A 9 MHz quartz crystal coated on both faces with an amount of porphyrin produced a frequency decrease of 21.4 kHz, which allowed ammonia in a 10.00 mL sample to be quantified in concentrations between 5 and 70 µg L^−1^, with a sensitivity of 0.60 Hz L µg^−1^, over a period of at least eight months. The proposed method has several advantages over the officially recommended indophenol spectrophotometric method: sample volume was reduced by a factor of 2.5, toxic reagents (phenol and sodium nitroprusside) were eliminated, analysing turbid samples presented no difficulty, and there was not only a significant time saving in solution preparation, but also in sample analysis time, which was reduced from 1 h to 2 min. No statistically significant differences (α = 0.05) were found both in the mean and precision of the results obtained for ammonia in water samples collected from domestic wells, analysed by this new method and by the indophenol spectrophotometric method. Furthermore, the proposed method would allow the individual quantification, with similar sensitivity, of amines and ammonia within a single analytical run.

## 1. Introduction

Ammonia is a key parameter in water quality control. Although natural sources have been responsible for most of its content in water, anthropogenic origins have greatly increased in the last century, and, presently, anthropogenic nitrogen fixation far exceeds the natural contribution [1]. The main anthropogenic sources are the fertilizers from the agriculture, sewage dumping from urban areas, and chemical plants. Although ammonia is an important source of dissolved nitrogen useful for plants, when in excess, it leads to excessive growth of biomass, upsetting the ecological balance of the water column. In spite of being a micronutrient, ammonia causes many adverse effects in living organisms. In humans, it is irritant to eyes, nose, throat, and lungs, and it corrodes mouth, oesophagus and stomach lining when ingested. According to the Portuguese legislation [2], ammonia concentrations in drinking water must not exceed 0.05 mg NH_4_^+^ L^−1^.

A variety of methods to quantify ammonia in waters can be found in the literature. The selection of the method must be made regarding the limits of detection and the concentration of ammonia in the samples, as well as the selectivity of the method in face of sample composition. Other figures of merit, such as sensitivity and precision, influence the choice of the method. Equipment and running cost, sample throughput, and the expertise needed in terms of personnel to run the analysis, also influence the selection of the method.

The spectrophotometric methods, based on the Nessler reagent, but most often on the Berthelot reaction, are the most widely used approaches to determine ammonia in water samples. Nesslerization is the oldest colorimetric method and it is based on the addition of Nessler reagent, an alkaline mixture of potassium iodide and mercury(I) chloride to the sample, and measurement of the absorbance at 425 nm [1]. It can only be used in wastewater analysis if errors of 1–2 mg L^−1^ are acceptable. The Berthelot reaction offers very high sensitivity, but the reaction involves relatively slow reactions between ammonia and phenol in the presence of hypochlorite to form indophenol blue in basic solution. Ammonia reacts with a hypochlorite to form a monochloramine, which reacts with phenol to form the intermediate quinone chloroimine. This intermediate reacts with a second molecule of phenol to form the indophenol chromophore that absorbs between 630 and 720 nm. Because it is a slow reaction, over an hour may be required, and nitroprusside is usually used as a catalyst. The complexities of the reaction have resulted in the extensive study of reaction conditions, pH, and temperature effects and the order of reagent addition [3,4,5,6].

Both colorimetric methods employ toxic reagents. The substitution of phenol by sodium salicylate requires increasing pH from 10.5 to 12.6, which worsens the risks of alkaline earth metal precipitation, and consequently turbidity. Besides the precipitation of dissolved metals, the presence of a few non-metallic elements as sulphur, selenium, and halogens, except for chloride, can also interfere in the indophenol blue method. More severe interference is expected from nitrogen compounds such as amines, amide, amino acid, nitrate, and proteins. The interference extension is dependent on the concentration of these species as well as on the specific version of the method used.

Fluorescence can also play an important role in the determination of trace concentrations of NH_3_. A fluorescence known method relies on the detection of the product of the reaction between NH_3_ and o-phthaldialdehyde. A known problem of this analysis is the higher responses obtained for seawater compared with the ones for freshwater.

In an attempt to determine ammonia with an inexpensive method and to avoid bulk instrumentation, several sensors have been developed in the past decades. Ammonia electrodes, which are in fact complete electrochemical probes with a pH electrode and a gas diffusion membrane, can be used if a base is added to the sample to increase pH. However, any gas able to cross the membrane and change pH would interfere, and volatile amines would give responses similar in magnitude to NH_3_. Moreover, the response is slow at low ammonia concentrations [7]. Polyvinylchloride (PVC) liquid membrane type electrodes have also been prepared using nonactin as ionophore. As selectivity of these nonactin membranes is poor [7,8], commercial mixtures with the trade name AI, containing 25% nonactin, have also been used as ammonium neutral carrier. The behaviour of these electrodes improves in an ionic medium, and near-Nernstian responses have been obtained in a Ca^2+^ background [8]. Solid electrolyte sensors using a thick film of NASICON and porous Cr_2_O_3_ operating at 350 °C [9], or a zirconium titanium phosphate cation exchanger membrane [10], were also reported. Selectivity could be improved adding a gas diffusion cell to prevent interfering ions from reaching the sensor.

Like electrochemical sensors, optical sensors based on pH indicator dyes and a gas permeable membrane are not selective for ammonia over basic gases from other origins. Several attempts have been made to turn the optical sensors insensitive to H^+^, by incorporating, for instance, the pH indicator in ormosils [11], or incorporating the H^+^ selective chromophore in nonactin PVC membranes [12]. Optical sensors based on the Berthelot reaction have also been reported; although, the devices cannot be considered true sensors, as they do not respond in a continuous way, due to the irreversibility of the reaction [13]. Nafion–crystal violet composite responds both to humidity and ammonia, but the reaction with ammonia is irreversible [14]. A dual layer sol–gel-based optical sensor for CO_2_ and ammonia was also reported, but only preliminary results for ammonia have been shown [15]. Tao et al. [16] succeeded in increasing sensitivity by bending the optical fibre, while Waich et al. [17] tried to eliminate the drawbacks associated with measuring fluorescence intensity, such as drifts in the optoelectronic setup, loss of light in the optical path, and problems found in the analysis of turbid samples, by the application of referencing methods. Porphyrins are known by their remarkable stability and their ability to interact with a wide range of compounds, including ammonia, while changing their characteristic absorption bands [18,19] or luminescence quenching [20].

Semiconductor metal oxide sensors usually show a slow response, incomplete recovery, and need high working temperatures. Besides SnO_2_, other oxides such as CuO, In_2_O_3_, Fe_2_O_3_, ZnO, TiO_2_, and WO_3_ have been used as sensing material [21,22,23,24]. Metals or additives have been added to enhance the sensitivity and selectivity of those materials [21,25,26]. Some metal complexes, such as potassium trioxalateferrate(III) [27], replaced metal oxides in an attempt to overcome the inconvenient of slow responses and incomplete recovery. Conductive polymers, such as polypyrrole [24] or polyaniline allowed working at room temperature [28,29,30,31]. Conductivity loss due to film aging could be overcome by incorporating carbon nanotubes [32]. Resistance changes in PANI films induced by ammonia have been enhanced with graphene [33], by preparing composite films such as PANI/Nb_2_CTx [34] (where Tx stands for some superficial functional groups such as -OH, -O, or F), or preparing PANI microspheres [35]. Depositing polyaniline on a flexible polyimide substrate allowed sensors to use in food material packages to be obtained [36]. Other resistance type sensors have been reported, such as nickel (II) macrocyclic complex, CuBr, or PbS based, and are mainly devoted to gaseous samples [37,38,39]. Surface acoustic wave (SAW) sensors can also be based on a change in conductivity, and, therefore, thin films of SnO_2_, ZnO, TiO_2_, and WO_3_, as well as polymers, have been applied in SAW devices [40,41].

Polyaniline nanoparticle films were also used to coat screen-printed interdigitated electrodes, assembled to a gas permeable membrane to create a headspace. Ammonia in gas phase could be determined by electrochemical impedance spectroscopy [42]; although, analysis is too long (28 min).

Both bulk and surface acoustic wave sensors based on gravimetric changes induced by ammonia, including in very few cases some elastic loading contributions, are another important group. These devices show responses that are proportional to the ammonia concentration, can work at room temperature, no reference is needed, especially for the bulk sensors based on commercial piezoelectric quartz crystals, and are easy to build and inexpensive. The measured parameter is frequency, which changes with the interaction between the recognition coating layer and ammonia. A large number of sensitive coatings have so far been used, from pyridoxine hydrochloride supported on a polymer, Ucon-75-H90,000 and Ucon-LBL-300X [43], extracts of Capicun annun seed pods [44], L-glutamic acid hydrochloride [45,46], polyvinylpyrrolidone [47,48], metal phosphonates [49,50], polypyrrole [50,51,52], a membrane with a 18-crown-6 ionophore [53], tri-diethylammonium salt of 4,4′,4″-[benzene-1,3,5-triyl-tr(ethin-2,1-diyl)tribenzoic acid [54], cryptophane [55], ZnO nanowires [56], nanofibrous membranes of poly(acrylic acid) and poly(vinyl alcohol) [57] to polyaniline [58], and I_2_ doped polyaniline [59].

Despite the large number of publications devoted to ammonia sensors, research is far from slowing down. Chemiresistive gas sensors are most often found in recent literature [60], and special materials such as graphene [60,61] and transition metal dichalcogenides [62] are among the most investigated. A quartz crystal resonator was recently employed in series with a PANI coated interdigital electrode [59]. The measurement of frequency brought significant advantages, namely a high shift while the resistance tuning effect was very weak. Ammonia flexible sensors are presently gaining special interest [60,61,62,63,64]. The search for the ideal sensor continues. Regeneration of the sensing layer is not always easy (it is for instance a problem with polypyrrole (PPY) films [21]), but selectivity, sensitivity, and lifetime are more general issues. However, those requests are defined by the application. Papers dealing with applications are scarce, and those with tests in rooms with manure [55] or in controlled ammonia concentration chambers for poultry [65] need to be mentioned. Ammonia in waste water was analysed by a nonactin-based potentiometric sensor [7], which only responded to ammonium ion, not to gaseous ammonia, and was strongly affected by the presence of alkali metals, especially by potassium. If the concentration of other cations, relative to ammonium, are not very high, a chemometric approach applied to measurements performed at two pH values was able to solve the interference problem [7].

Sensors need to be developed for a particular purpose, not only because information on matrix composition is crucial, but also because the complete method for the analysis needs to be established, taking in consideration the properties of the sample.

The objective of this work was the development of a reliable expeditious method to quantify ammonia in freshwaters. The method relies on a coated piezoelectric quartz crystal, with a frequency sensitivity for ammonia adequate for the purpose.

The sensor will be based on a coated quartz crystal. These sensors have some advantages over the electrochemical sensors, and one of them is that coating conductivity is not an issue. Compared to potentiometric sensors, there is no need to adjust ionic strength of solutions, and response dependence on concentration is not logarithmic.

By careful choosing the chemical to coat the quartz crystal, it is possible to obtain good chemical stability, sensitivity to ammonia, and also selectivity, which means a strong preference for ammonia over other species present in the sample, which is often a major problem with metal oxide semiconductors. It is however preferred not to have a very strong interaction between coating and the analyte, meaning covalent bonds should be avoided, as the sensor needs to be reusable, and, therefore, reversibility is mandatory. Porphyrins have been used on sensors, based on several transducer principles [66], namely, on piezoelectric devices [67]. Morales-Bahnik et al. [68] have reposted the changes in absorbance produced by ammonia solutions on metal 5,10,15,20-tetra(phenyl)porphyrin (TPP) immobilized on nitrocellulose. After experiments with metalloporphyrins, with different central atom (Co(II), Ni(II), Cu(II), Sn(II), Hg(II), or Mn(II)), they have found that the Mn(II) complex showed the highest sensitivity to ammonia. Therefore, for the present work, a few Mn(II)-related porphyrins were synthesized and tested on the gold electrode of a piezoelectric quartz crystal, in order to select the most sensitive coating for ammonia.

Quartz crystals are piezoelectric and vibrate when an oscillating electric field is applied between the two metal electrodes, one on each face. The frequency of oscillation is dictated by the thickness of the quartz thin wafer. Surface acoustic wave sensors also use piezoelectric materials, but the acoustic wave does not propagate in the bulk of the material, rather only in its surface.

Sauerbrey [69] was the first to state that the deposition of a film of mass M (g) on the active area A (cm^2^) of a piezoelectric quartz crystal oscillating after the application of an alternating potential difference, decreases its frequency of oscillation F (MHz) by ΔF (Hz):

ΔF = −2.3 × 10^6^ F^2^ (ΔM/A)

This relation, between frequency decrease and mass, is the reason to call these sensors quartz crystal microbalances. This equation is, however, only valid for thin rigid films uniformly distributed over the active area. For this reason, it is generally used to estimate the amount of the coating film, but not for quantitative analysis. In addition, there may be other contributions to frequency [70], such as, for instance, the mechanical aperture of the crystal in the cell, and it is mandatory to use calibration with standards, such as with any other instrumental technique.

Most applications are based on gravimetric changes, but these sensors may be much more than mass sensors [70], which is not important for the present application, but dictates the preference for designations such as bulk acoustic wave sensors or thickness shear mode sensors.

We present not only a sensor for ammonia, but an analytical methodology designed to quantify ammonia in freshwater. The main goal was not on continuing the search for improving the limit of detection of the ammonia sensor, but rather to have a method covering the range of concentrations found in the samples, including the maximum allowed limit. Selectivity cannot be forgotten and must be addressed, in the context of the freshwater analysis. This new method detects ammonia in gas phase, meaning that only volatile compounds reach the sensor, and non-volatiles are no longer of concern. Non-hazardous, simplicity, cost, and celerity of the method, allied to sensor durability and reliability were the main sought characteristics.

In order to evaluate the reliability of the new method, the results of the analysis of water collected from domestic wells were compared with the results obtained using the established spectrophotometric method based on the indophenol method and Berthelot reaction.

## 2. Materials and Methods

### 2.1. Reagents

A standard stock ammonium solution 1000 mg L^−1^ was prepared by dissolving the appropriate amount of dried ammonium chloride p.a. (Merck 101145) in MilliQ water. Standards were prepared by appropriate dilutions of the stock ammonium solution in Milli-Q water. Sulphuric acid (Merck 100731) and sodium hydroxide (Panreac 481934) were used to adjust pH of the water samples.

- Reagents used in the sensor coating and testing

**Figure 1:**
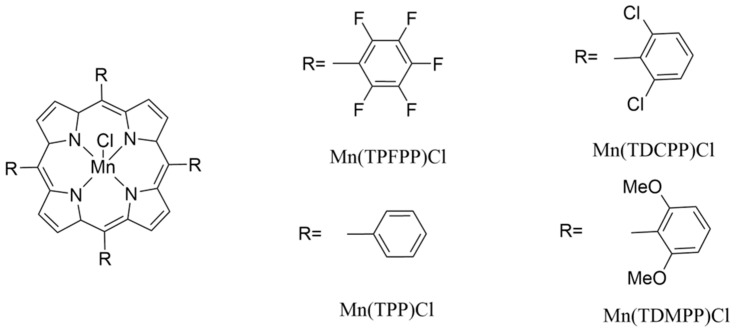
Structure of the metalloporphyrin related structures, used to coat the piezoelectric quartz crystal.

The metalloporphyrin derivatives, whose structures are shown in Figure 1, were synthesized according to the literature [71,72,73]. Each one was dissolved in chloroform p.a. (VWR 22711.324), and used to coat the quartz crystal.

Ammonia gas, for testing coating sensitivity, was withdrawn from a cylinder (PRAXAIR).

- Reagents used in the indophenol method

A phenol solution was prepared by dissolving 10 g of phenol (Panreac 131322) in 100 mL of 95% ethanol (Merck 100983). The sodium nitroprusside solution (0.5% *w*/*v*) was prepared by dissolving 0.5 g of sodium nitroprusside (RPE 481934) in 100 mL of Milli-Q water. The alkaline citrate solution was prepared dissolving 100 g of trisodium citrate (José M.Vaz Pereira UJ740) and 5 g of sodium hydroxide (Panreac 481934) in 500 mL of Milli-Q water. Oxidizing solution was prepared mixing 100 mL of sodium citrate solution with 25 mL hypochlorite solution (Panreac 212297).

### 2.2. Apparatus

#### 2.2.1. Apparatus for Coating

The piezoelectric crystals were polished AT-cut HC-6/U with gold electrodes, with a resonance frequency at 9 MHz (ICM-International Crystal Manufacturing Co, Inc., Oklahoma City, OK, USA).

**Figure 2:**
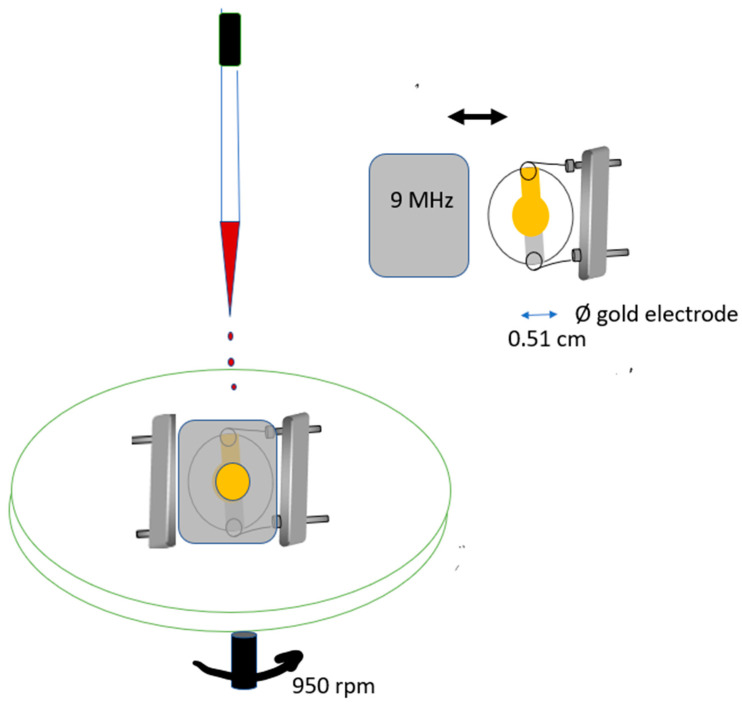
Picture of the bare quart crystal, out of its metal lid, and scheme used for coating application: small drops of the porphyrin solution were applied over the crystal electrode, through a hole cut at the centre of the lid, and spread by spinning.

A spin coater (Süss Delta 10 BM) was used to coat the piezoelectric quartz crystals. Figure 2 shows a piezoelectric quartz crystal out of its metal lid. A circular hole was cut on the lid, so that the coating solution could be applied. Coating was spread by spinning the quartz crystal inside its lid. To guarantee good balance, another pair of contacts was soldered on the top of the lid. Coating was applied over the electrodes on both faces, the lid was taken out, and the crystal was left to dry. The amount of coating was calculated applying the Sauerbrey equation where ΔF was the difference between the frequency of the coated crystal and the frequency of crystal before coating, read under the same conditions.

#### 2.2.2. Apparatus for Coating Selection

**Figure 3:**
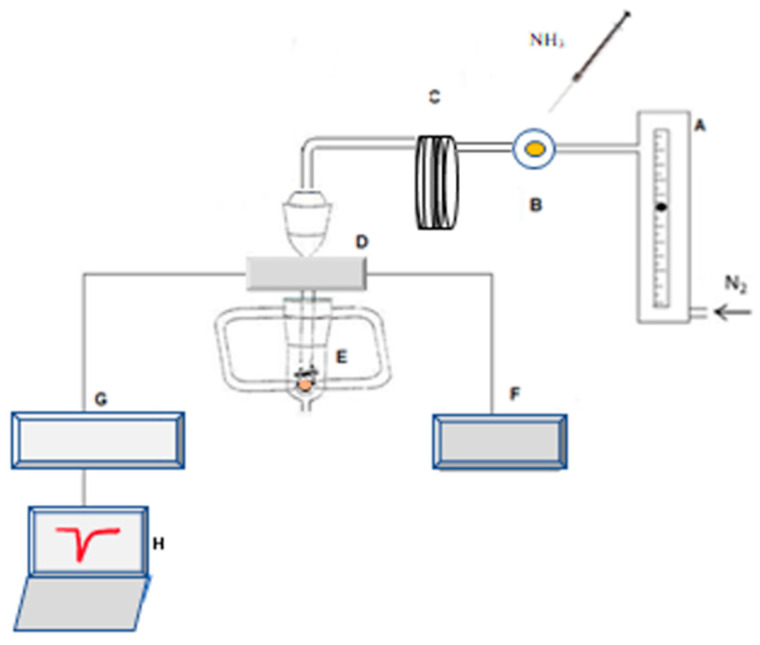
Experimental layout for coating selection: A—flowmeter; B—injection port; C—coil; D—oscillator; E—crystal cell; F—power source; G—frequency meter; H—personal computer for data acquisition.

Figure 3 shows the experimental layout used to evaluate the diverse metalloporphyrin molecules for ammonia sensing. Precise volumes of NH_3_ were injected with a gastight syringe in an Omnifit injection valve, and carried by nitrogen flow to the cell housing the coated crystal. The quartz crystal was connected to a home-make oscillator connected to a power supply. The frequency of oscillation of the crystal was monitored with a frequency counter Leader 823A and recorded on a PC, with data acquisition software written in Lab View.

The height of the inverted frequency (decrease) peak observed after the ammonia injection was recorded.

A flowmeter assured that a constant nitrogen stream was flowing through the experimental setup and reached the quartz crystal. Precise volumes of NH_3_ were injected on an injection port by a gas-tight syringe and carried by the nitrogen flow to the crystal cell. The tube connecting the injection port to the crystal cell was longer than necessary to make the connection, so that the gaseous ammonia was delayed a few seconds in order to separate the analytical signal from a small pressure signal that may occur, due to the pressure perturbation caused by the injection. The tube was coiled, a practice common in flow injection analysis that allowed the induction of secondary flow, reducing axial dispersion.

#### 2.2.3. Apparatus for the Analysis of Aqueous Standards and Water Samples

**Figure 4:**
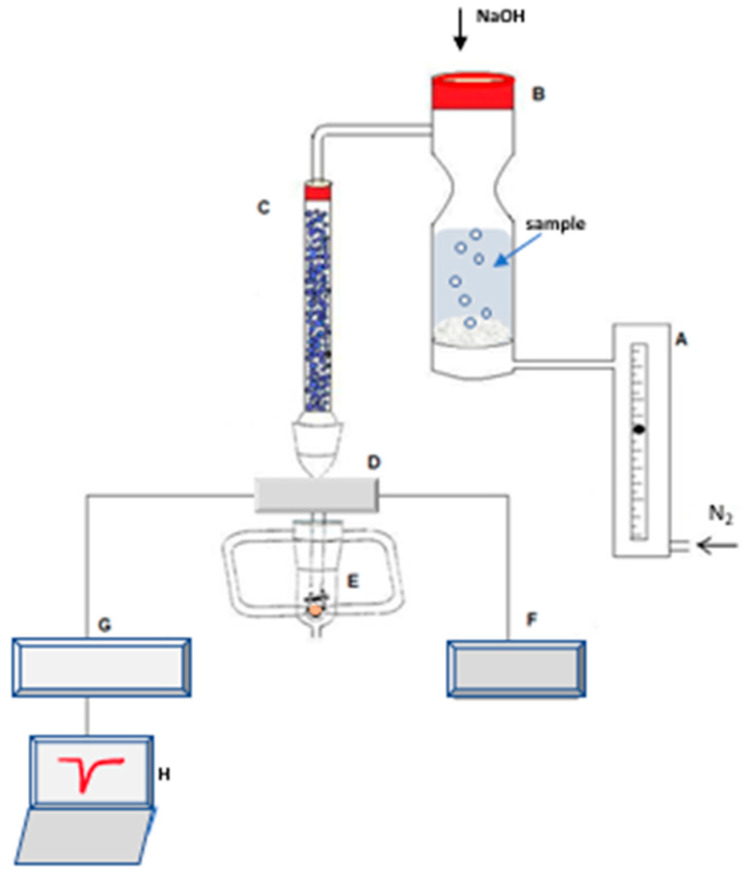
Experimental layout: A—flowmeter; B—sample cell; C—tube with silica gel; D—oscillator; E—crystal cell; F—power source; G—frequency meter; H—personal computer for data acquisition.

Figure 4 shows the experimental layout used to quantify ammonia in water samples. A sensor based on a piezoelectric quartz crystal coated with the chloro[5,10,15,20-tetrakis(pentafluorophenyl)porphyrinato] manganese(III), Mn(TPFPP)Cl was used for ammonia detection. The quartz crystal was connected to a home-make oscillator connected to a power-supply. The frequency of oscillation of the crystal was monitored with a frequency counter Leader 823A and recorded on a PC, with data acquisition software written in Lab View.

A sample glass cell, with a sintered glass plate and a nitrogen entrance at the bottom, was used to accommodate the sample to be analysed. An accurately measured volume of the sample was introduced by opening the septum cap on top of the cell. The septum was later used to inject the NaOH solution, used to convert ammonium into gaseous ammonia (NH_4_^+^ + OH^−^ = NH_3_ + H_2_O), which was carried by the nitrogen stream to the quartz crystal.

In order to increase sensitivity, the nitrogen stream carrying the ammonia gas was divided in two, and directed to both coated faces of the quartz crystal.

### 2.3. Procedure

#### 2.3.1. Sample Collection and Preservation

Water samples were collected from seven wells and transported to the laboratory in bottles filled till the exclusion of headspace.

In the lab, they were filtered through MSI^®^ acetate plus filters with porous of 0.45 µm. After filtration, samples were acidified to pH 2 with H_2_SO_4_, and cold preserved up to 4 °C, in the dark [1].

#### 2.3.2. Crystal Coating

The piezoelectric quartz crystal was coated by applying a drop of the Mn(TPFPP)Cl metalloporphyrin solution on the centre of the electrode. The drop was spread by spinning the quartz crystal. Both sides were coated. The coated crystal was then allowed to dry for 48 h. After solvent evaporation, the frequency decrease due to coating was registered. A frequency decrease of 21.4 kHz, corresponding to 23 μg, was obtained.

#### 2.3.3. Analysing Standards and Samples

Nitrogen was turned on, and flow adjusted to 50 mL min^−1^.

An accurate volume of 10.00 mL of the sample or of an ammonium chloride standard solution was placed on the glass cell by opening the cap on its top. After closing the cap, nitrogen begins to reach the quartz crystal and its frequency of oscillation soon stabilizes. Sodium hydroxide solution (1 mL) was then injected into the cell to raise pH to 11, assuring that ammonium ion was converted to ammonia. The nitrogen flow carried the ammonia gas to the quartz crystal cell, and the frequency of the quartz crystal began to decrease as soon as the NH_3_ interacted with the crystal coating. The difference in the frequency of the crystal before the NaOH injection (baseline), and the minimum value observed after its introduction was computed. Afterwards, frequency increased and reached the baseline value in less than 2 min, which indicates the complete recovery of the sensor. No new sample, or standard, was analysed before baseline frequency was attained.

## 3. Results and Discussion

**Figure 5:**
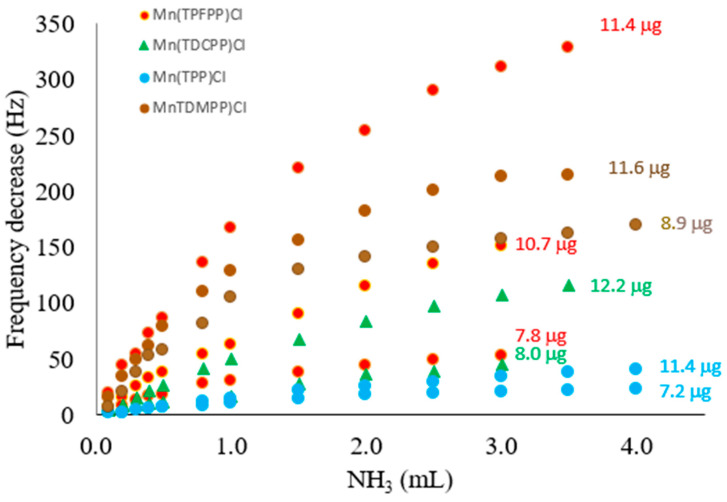
Calibration curves obtained with quartz crystals coated with the different porphyrins. Coating amounts on the electrodes of each crystal are shown.

Figure 5 shows the calibrations plots (frequency decrease vs. volume of NH_3_ injected) obtained with crystals coated with the four different metalloporphyrins. Placed on the right of each curve, the coating amount can be seen, calculated using the Sauerbrey equation.

As the coatings can be considered as pretty rigid solids, uniformly distributed on the crystal active surfaces, Sauerbrey equation was used to calculate the coating amount from the frequency decreases observed after the coating was applied and left to dry. For the same coating compound, it can be seen that responses increase with the coating amount. Mn(TPP)Cl is the less sensitive compound as can be seen looking at the second blue curve, counting from the bottom, to which corresponds the biggest coating mass, and also the biggest number of porphyrin molecules. The Mn(TDCPP)Cl comes next in sensitivity, followed by Mn(TDMPP)Cl. The most sensitive porphyrin was Mn(TPFPP)Cl, and, therefore, it was the chosen to coat the ammonia sensor.

**Figure 6:**
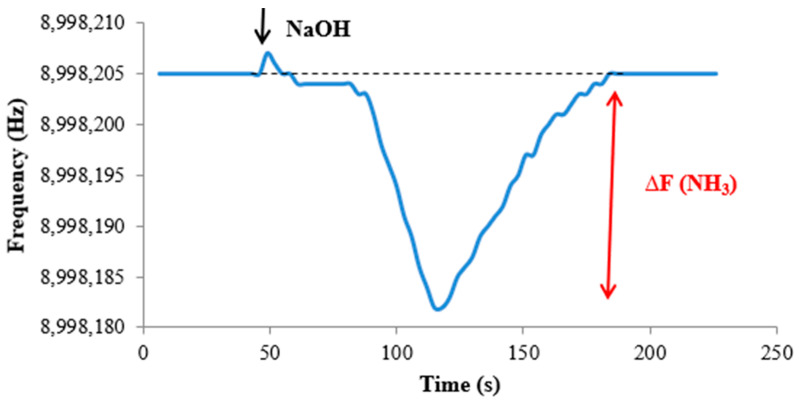
Frequency of the sensor during the analysis of a 30 µg L^−1^ ammonium chloride standard solution (dashed grey line shows baseline frequency).

Figure 6 shows the frequency of a quartz crystal coated with Mn(TPFPP)Cl, after the introduction of 10.00 mL of a solution of NH_3_ 30 µg L^−1^. After the NaOH injection, ammonium ion was converted into gaseous ammonia, which was carried by the nitrogen flow to the quartz crystal cell. After reaching the coated quartz crystal, frequency starts to decrease, and the difference between baseline frequency and the minimum is computed. As can be seen, the frequency decreased 23 Hz after NaOH injection.

There are a series of advantages in performing the experiments under a constant inert flowing gas. One of them is that there is no need for a long baseline frequency stability, as the analytical response is computed starting from the frequency value before the frequency starts decreasing, due to ammonia. Frequency stability better than ±1 Hz must be assured for a limited period of time, enough to evaluate this condition and decide if it is safe to start the analysis, and for the time it takes to be seen at the minimum of frequency (peak). Analysis time is very short, as there is no need to wait for equilibrium. The response of the sensor to a particular ammonia concentration is reproducible, as long as the nitrogen flowing rate is maintained.

This frequency decrease was proportional to the NH_3_ concentration, and a linear calibration graph was obtained between 5 and 70 µg L^−1^, with a sensitivity (slope) of 0.60 Hz L µg^−1^. The used coating amount, which produced a frequency decrease of 21.4 kHz, and solution volumes of 10 mL, was therefore shown to be adequate for the desired application. These values were selected after an optimization process, and significantly lower values of frequency decrease due to the coating, obtained after coating spreading and solvent drying, on the two faces of the quartz crystal, were found to be inadequate for the present application. Significant larger coating amounts, would allow decreasing sample volumes, but were difficult to apply without impairing crystal vibration.

Responses to NH_3_, depending on the magnitude of the frequency decrease, were obtained, typically, in about 70 s after NaOH injection, and sensor reversibility was found to be complete after another 70 s. Therefore, although the time that elapsed from the NaOH introduction to complete recovery of the sensor was dependent on the peak magnitude, it was to be around 2 min (average).

Besides being completely reversible (another analysis could not be started if the frequency had not yet reached the baseline value recorded before the NaOH injection) and able to give a response in a very short time, this new sensor shows a remarkable stability, which is translated in the maintenance of calibration sensitivity, which assures a long sensor lifetime. Table 1 shows a few calibration lines obtained within a period of eight months. Sensitivity of the sensor did not show any degradation within the eight-month period. A degradation in sensitivity would result in a limitation in the sensor capacity to differentiate samples with close concentrations, and would dictate the end of the sensor life, as recalibration could solve this issue. Such a remarkable chemical ruggedness is seldom found in sensors [74].

The experimental layout used in this work was based on the detection of gaseous ammonia; although, the sensor does also show very good stability in contact with water solutions. The detection in the gaseous phase was preferred in order to reduce the number of possible interference compounds able to reach the sensor. Even so, volatile compounds are prone to interfere, and amines are the main group of concern. Diethylamine and glycine are two amines usually analysed in freshwater [1] and were therefore added to ammonia solutions of known concentration, in order to test for possible interferences in the proposed method.

**Figure 7:**
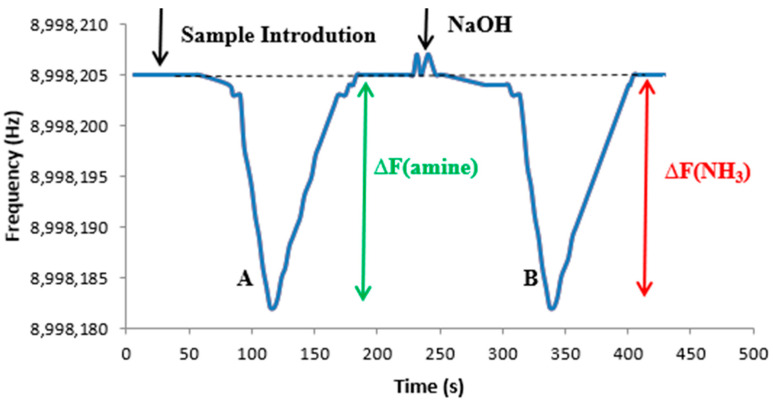
Frequency of the sensor during the analysis of a solution 1.8 µmol L^−1^ in ammonia and 1.8 µmol L^−1^ in glycine (dashed grey line shows baseline frequency).

Figure 7 shows the frequency of the coated crystal when a solution of 1.8 µmol L^−1^ (30 µg L^−1^) in ammonia and 1.8 µmol L^−1^ in glycine was analysed. After the introduction of the solution into the cell, the frequency started to decrease as the amine began to reach the coated crystal, and, after 80 s, the minimum frequency was reached (peak A). Then, the frequency of the crystal started to increase to its initial value, indicating full desorption of the amine from the electrode surface. NaOH was then injected and the frequency shift corresponding to NH_3_ could be observed, without interference of the amine. Peak B corresponds to the signal of ammonia, which appears clearly separated from the amine signal. Diethylamine behaviour was very much similar to the one described for glycine.

Peaks A and B are of equal magnitude, which shows that the sensor detected both amines and ammonia with the same molar sensitivity. Therefore, this method could be used to independently quantify both amines and ammonia within a single sample running.

This new method based on a porphyrin coated sensor was used to determine the ammonia concentration in freshwater. The freshwater was collected from seven wells located in two geographic areas located in the centre of Portugal, one on the littoral, Aveiro district (A), and the other one 90 km inland, in the Viseu district (V). Within the same district, the minimum distance between wells was 2 km and the maximum 30 km. Five replicates of each sample were analysed, both by the sensor method and by the indophenol method; 10.00 mL or 25.00 mL of each sample was used in the sensor and indophenols methods, respectively.

Table 2 shows the ammonia concentration found for each sample. A and V prefixes identify the Aveiro and Viseu districts, respectively. Samples from two wells in the Aveiro district and five wells in the Viseu district were analysed.

Comparing the results obtained by the sensor and indophenol method, by performing a paired *t*-test and an F test, no significant difference (α = 0.05) in the media of the replicate values, nor in the precision of the results obtained by both methods, were found.

## 4. Conclusions

The new method is based on a remarkably long-lasting sensor that maintains sensitivity for at least eight months. The application of the new analytical system to the analysis of ammonia in freshwater samples allowed results that are not statistically different from the ones obtained by the indophenol spectrophotometry to be found. Moreover, the new sensor method shows several main advantages over the indophenol method: the indophenol method uses a larger volume of sample, and uses many more reagents, some of them highly toxic, such as phenol and sodium nitroprusside; oxidizing solution needed to be prepared each day, and colour development in each standard and sample took one hour, which made the analysis by the indophenol method tedious and time consuming.

## Figures and Tables

**Table 1:** Calibration lines obtained for the same sensor, along 8 months.

Date	Calibration Line
26 May	−ΔF = 0.599 [NH_3_] − 1.455
8 December	−ΔF = 0.608 [NH_3_] − 0.746
6 January	−ΔF = 0.609 [NH_3_] + 0.453
25 January	−ΔF = 0.607 [NH_3_] + 0.364
1 February	−ΔF = 0.612 [NH_3_] + 1.104

**Table 2:** Results obtained analysing samples using the acoustic sensor and the indophenol method.

Sample		A1	A2	V1	V2	V3	V4	V5
NH_3_ (µg L^−1^)	Sensor	42.6 ± 1.4	20.1 ± 1.4	20.7 ± 1.3	20.4 ± 1.5	25.1 ± 1.9	22.0 ± 1.4	29.7 ± 1.8
	Indophenol Method	42.3 ± 1.3	20.0 ± 1.7	20.7 ± 1.5	20.4 ± 1.6	25.9 ± 1.6	21.9 ± 1.7	29.6 ± 1.5

## Data Availability

Not applicable.
